# Breaking the Taboo: Understanding the Relationship between Perception, Beliefs, Willingness to Eat Insects, and Food Neophobia among Polish Adults

**DOI:** 10.3390/foods13060944

**Published:** 2024-03-20

**Authors:** Julita Szlachciuk, Sylwia Żakowska-Biemans

**Affiliations:** Department of Food Market and Consumer Research, Institute of Human Nutrition Sciences, Warsaw University of Life Sciences, Nowoursynowska 159c, 02-776 Warsaw, Poland; julita_szlachciuk@sggw.edu.pl

**Keywords:** insects, beliefs, novel food, alternative protein sources, food neophobia

## Abstract

Insects are a potential source of protein for direct human consumption or indirectly in processed foods. The research examined consumer perceptions and beliefs regarding the consumption of insects and food products containing insect proteins. The study aimed to identify beliefs about insect consumption, assess the perception of food products containing added insect proteins, and gain a deeper understanding of the role of food neophobia in accepting insects as an alternative source of protein. The data were collected in 2023 through a cross-sectional quantitative online survey of a group that was representative of consumers by age, gender, and region sample (*n* = 1000) who were responsible for food purchasing decisions in their households. While many respondents viewed foods enriched with insect protein as innovative and environmentally friendly, others found the idea of consuming insects repulsive and expressed concerns about potential allergic reactions. Food neophobia was a determining factor in respondents’ beliefs and perceptions regarding the consumption of insects and food products containing insect proteins. Respondents were more inclined to consume products with added insect protein rather than visibly identifiable insects. The results highlight the need for targeted marketing strategies and product development approaches to align with the varied expectations of consumers in the rapidly expanding insect-based food market.

## 1. Introduction

Many cultures, especially in Africa, Asia, and Latin America, have a long history of incorporating insects into their diets [1]. In these regions, specific insect species are often considered delicacies and are prepared in various ways, e.g., fried, roasted, or ground into powders [2]. While some edible insect species, such as grasshoppers and locusts, require the removal of legs and wings prior to consumption, many edible insect species can be consumed whole but may also be processed into powder or paste [3].

Insects are nutritionally dense, offering a rich source of essential amino acids, healthy fats, vitamins, and minerals [4,5,6]. The composition of omega-3 polyunsaturated and some other fatty acids in mealworms is comparable to that found in fish and higher than that in pigs and cattle [7]. They are also considered a good source of dietary fiber [8]. Insect proteins represent a compelling alternative protein source that addresses critical issues such as nutritional deficiencies, environmental sustainability, and global food insecurity [1].

Compared to conventional livestock, insect farming is exceptionally sustainable. Insects are highly efficient in converting feed into protein, requiring significantly less land, water, and feed [9]. They produce fewer greenhouse gas emissions and contribute minimally to deforestation. Furthermore, insect farming can utilize organic waste materials, creating a closed-loop system that reduces environmental impact [10].

In many Western societies, there is a cultural aversion to consuming insects. In these regions, insects are notably absent from mainstream diets, relegating their presence to the realm of novelty snacks within the niche food sector [3]. The reluctance of consumers in Western Europe to accept insects as a food source can be attributed to a combination of cultural, psychological, and sensory factors [11,12,13]. While entomophagy has the potential to address food security and sustainability issues, challenges such as cultural acceptance, regulatory frameworks, and consumer perceptions need to be addressed for widespread adoption in regions where eating insects is not a traditional practice. Another issue to be addressed pertains to the potential risks associated with incorporating insect powder into food, encompassing concerns related to allergenicity and the environment, as well as chemical and biological hazards, which may adversely impact consumer perception [14,15]. There is a noticeable trend indicating increased consumer acceptance of utilizing insects as animal feed; however, the degree of acceptance varies depending on the species of the animal in question [16,17]. Finally, it was observed that people in Western culture are more willing to accept food products containing insects rather than the whole insect [18]. There has been an increasing interest in incorporating insect powder into common food products like snacks, baked goods, and pasta to enhance their protein content [19,20].

The results of review studies indicated that the main motivations that determine the consumption of edible insects are related to gender, age, sustainability, nutritional value, sensory attributes, tradition/culture, food neophobia, disgust, and familiarity/past experiences [18,21]. Men were more willing to try eating insects than were women [22,23,24].

Research into the acceptance of insects and other innovative foods by consumers typically emphasizes psychological factors influencing the initial willingness to eat [25]. Food neophobia has been found to be an important barrier to the consumption of insects [23,26,27]. High levels of both food neophobia and disgust, which have been shown to be strongly associated, may hinder willingness to consume edible insects, particularly due to a lack of familiarity with the product itself [28]. Neophobia is also linked to sensory expectations about food. Insects may be perceived as having an unpleasant taste or texture, deterring individuals from trying them [22]. A study by Ordoñez López et al. [29] indicates that the difficulties associated with accepting insect-based foods can be effectively addressed through repeated exposure to entomophagy, which reduces food neophobia and food disgust sensitivity. Embracing the consumption of insects requires a shift in cultural perceptions, but the potential benefits in terms of health, sustainability, and economics make them a viable option for the future.

Therefore, the main aims of the study were threefold: (1) to identify beliefs about consuming insects, (2) to assess perceptions of food products containing added insect proteins, and (3) to gain further insights into the role of food neophobia in the acceptance of insects as an alternative protein source.

## 2. Materials and Methods

### 2.1. Participants

A total of 1000 participants were recruited from a specialized online access panel owned by a professional market research company, SW Research, conducting studies in accordance with the standards set by the European Society for Opinion and Marketing Research (ESOMAR) International Code on Market, Opinion, and Social Research, as well as in compliance with the General Data Protection Regulation of the European Union (GDPR, Regulation 2016/679). The study was conducted according to the ethical principles expressed in the Declaration of Helsinki. A nationwide sample was obtained by applying selection criteria that matched the population distributions in Poland for gender, age, place of residence, and region. Stratified random sampling was employed based on these criteria. Only individuals responsible or co-responsible for decision-making and food purchases in their households were eligible to participate in the study. Informed consent was obtained from respondents before the collection of the survey data. Before commencing the study, participants were informed about the nature of their involvement and their rights. In order to begin responding to the questionnaire, participants were required to tick a box indicating their understanding of the study’s overall objective and their agreement to participate in the online survey. The collected data were encoded in a non-identifiable format and processed anonymously.

### 2.2. Questionnaire Structure

The questionnaire consisted of three primary sections commencing with single-choice questions addressing participants’ attitudes toward purchasing new food products. Participants were asked to choose the statement that best described their attitude toward buying new food products, with options ranging from being the first among friends to buy to expressing reluctance to buy new food products. The subsequent questions focused on participants’ awareness of edible insects. They were asked about their familiarity with the concept of consuming insects and about the consumption of insects or insect-based food.

The second part of the questionnaire consisted of a set of questions about beliefs regarding eating insects, perception of food with added insect proteins, food neophobia, and willingness to eat insects, including food products containing insects in processed form. The beliefs about insects were measured based on items developed by Ruby and Rosin [30]. Items were used to measure the extent to which participants believed eating insects is beneficial, risky, and disgusting. Participants were provided with a set of statements about eating insects in random order, using the seven-point interval scale: “strongly disagree” (1); “disagree” (2); “somewhat disagree” (3); “neutral” (4); “somewhat agree” (5); “agree” (6); “strongly agree” (7). These items covered the benefits of eating insects (“Rearing insects for food generates less pollution and greenhouse gas than rearing conventional livestock”; “Rearing insects as food is more efficient and requires fewer resources than rearing conventional livestock”; “Insects are highly nutritious”) and the disgustingness of eating insects (“The idea of eating insects makes me nauseous”; “I am offended by the idea of eating insects”; “Eating insects is disgusting”). The risk associated with eating insects was measured with items such as the following: “Insects carry harmful microbes”; Insects contain harmful toxins”; “Eating insects will increase the risk of infectious disease”; “Eating insects would expose me to harmful chemicals and insecticides”; “Some people would have allergic reactions to eating insects”.

Participants’ perceptions regarding the consumption of food products with added insect proteins were assessed using a semantic differential scale. They were asked to rate their agreement or disagreement with statements concerning the perceived health benefits, environmental impact, naturalness, acceptability, safety, nutritional value, cost, protein content, trustworthiness, and innovativeness of consuming insects. Each statement was anchored with one end denoting a positive attribute of insect consumption (such as being beneficial to health, environmentally friendly, natural, acceptable, safe, high in nutrition value, etc.) while the other end represented a negative attribute (like being unfavorable for health, non-environmentally friendly, unnatural, unacceptable, unsafe, low in nutrition value, etc.). To mitigate order effects or biases, each item was presented in different orders to various respondents. Participants were instructed to indicate their level of agreement or disagreement with each statement using a seven-point scale. The questionnaire was prepared and presented to the respondents in Polish. Food neophobia was measured using the scale developed by Pliner and Hobden [31]. Over the past years, this scale has been utilized in numerous studies examining consumer responses to edible insects as food [21,28]. All 10 items were assessed on a seven-point scale, ranging from “Completely disagree” to “Completely agree”.

To assess consumers’ willingness to eat insect-based food, respondents were asked the extent to which they would be willing to eat the following products: whole visible insects (e.g., fried insects); whole insects, but not visible (e.g., insects in chocolate); ground insect powder (e.g., insect powder in bread; isolated insect protein (e.g., insect protein in a protein bar)). The questions were formulated using a seven-point interval scale from one (definitely unwilling) to seven (definitely willing). Additionally, a question was included to allow respondents to indicate that they are not willing to eat products containing insects at all: “I am unable to eat a product that contains insects”. Finally, socio-demographic characteristics, such as age, gender, education level, place of residence, income, and subjective assessment of the financial situation, were included in the questionnaire.

### 2.3. Data Analysis

Data were analyzed using descriptive statistics, parametric (ANOVA) and non-parametric tests (chi-square), and multivariate analysis (factor analysis). Means, standard deviations (SD), and frequencies (%) were calculated using descriptive analysis. Among others, descriptive statistical analysis was used to describe the socio-demographic characteristics of the sample and characterize it regarding food neophobia. The chi-square test was performed to identify significant differences between the selected variables.

Food neophobia scores (FNS) were calculated based on the sum of responses for the 10 FNS statements, with a possible score range of 10–70. A higher score indicates a greater degree of food neophobia. Based on the food neophobia scores, participants were divided into tertiles to create three food neophobia groups that were termed “neophilic” (low), “neutral” (medium), and “neophobic” (high). This grouping method is consistent with previous studies [32,33,34]. Participant characteristics among food neophobia groups were compared using the chi-squared test and ANOVA with post hoc test (Scheffé’s method).

Exploratory factor analysis (EFA) was also used to analyze the results. The extraction method used in factor analysis was principal component analysis, and we used factors with an eigenvalue greater than 1. Sources of information with factor loadings of at least 0.50 were included. Varimax rotation was performed to facilitate the interpretation of each factor. The factorability of the data was confirmed using the Kaiser–Meyer–Olkin (KMO) measure of sampling adequacy and Bartlett’s test of sphericity (*p* < 0.001). Cronbach’s alpha coefficient was calculated to check the reliability of the factor analysis.

The collected data were analyzed using the IBM SPSS Statistics v. 29 statistical software package, Armonk, NY, USA: IBM Corp.

### 2.4. Characteristics of the Sample

More than half of the respondents were female (53.0%) (Table 1). Most respondents were 60 years old and older (27.1%), while more than one-fifth fell into the 18–29 age group. Over 19% of the respondents were aged 30–39, and those aged 50–59 constituted 17.5%. The smallest group of study participants consisted of individuals aged 40–49. Considering educational background, the majority of respondents reported having secondary education (48.1%). More than 40% of respondents claimed to have higher education. The smallest number of study participants indicated having primary education (2.1%) and vocational education (9.4%). More than half of the respondents were employed full-time. Retired individuals constituted 21.7% of the study participants, while less than 8% were students. Over one-third of the study participants declared their income in the PLN 5001–9000 range. More than 29% of respondents stated that their household income ranged from PLN 1001 to PLN 5000. Household income below PLN 1000 was reported by 4.3% of respondents. More than 15% of study participants declined to answer this question. When asked about their subjective assessment of their financial situation, over 60% of respondents answered, “I live sparingly to save money for bigger expenses”. Less than one-fifth of the survey participants stated, “I have enough money for everything without special savings”. Over 11% mentioned, “I live sparingly and have enough money for my basic needs”. Respondents were least likely to choose the answers “I am wealthy” (2.0%) and “I do not have enough money for my basic needs” (2.1%). In terms of household composition, the study participants primarily lived in two- and three-person households (27.5% and 27.3%, respectively). The smallest percentage of respondents lived in single-person households (11.3%). Over 40% of respondents resided in rural areas, and 31.7% lived in cities with more than 100,000 inhabitants. The smallest percentage of respondents lived in cities with more than 500,000 inhabitants. Additionally, over 47% of respondents declared they have no children under 18 in their households.

## 3. Results

### 3.1. Food Neophobia

The mean FNS score of the respondents was 39.92 (SD = 10.126). The cut-off points were at one standard deviation from the mean. Participants were divided into three groups based on their food neophobia score: low (10–29.8; N = 124), medium (29.9–50.05; N = 740), and high (50.06–70; N = 136). Respondents with a low score were assigned to the “neophilic” group, which accounted for 12.4%. Study participants who scored on average were assigned to the “neutral” group (74.0%). The respondents with the highest score accounted for 13.6% and were assigned to the “neophobic” group. Participant characteristic distributions among food neophobia groups did not significantly differ in place of residence. They significantly differed in gender, age, education level, household income, assessment of the financial situation, number of people in the household, and children under 18 years old in the household (Table 2).

Taking gender into account in the analysis, it can be seen that a significantly higher level of neophobia occurs among women (M ± SD = 39.96 ± 10.916). Further comparison of the food neophobia score showed that it increases with age and is significantly higher in people aged 60 and over (M ± SD = 43.85 ± 11.369). As the level of education increases, the level of neophobia decreases significantly; the same is observed in the case of an increase in income level in the household. The lowest food neophobia level is recorded in households declaring income above PLN 9000. PLN (M ± SD = 38.21 ± 10.969). The highest food neophobia level was recorded in one-person households (M ± SD = 43.18 ± 11.661) and the lowest in four-person households (M ± SD = 43.18 ± 11.661). The level of neophobia is also lower in households with children under 18 years of age (M ± SD = 38.00 ± 9.351). People living in rural areas show a higher level of neophobia (M ± SD = 40.38 ± 9.364), but this result is not statistically significant (Table 2).

### 3.2. Previous Experience with Insects

More than half of the study participants admitted that they had never eaten insects or insect-based foods (67.6%). Just over 11% of respondents had eaten insects or insect-based food once. About 5% of the study participants had eaten insects or insect-based foods more than once. Over 16% of those surveyed were unsure if they had eaten this type of food.

**Figure 1:**
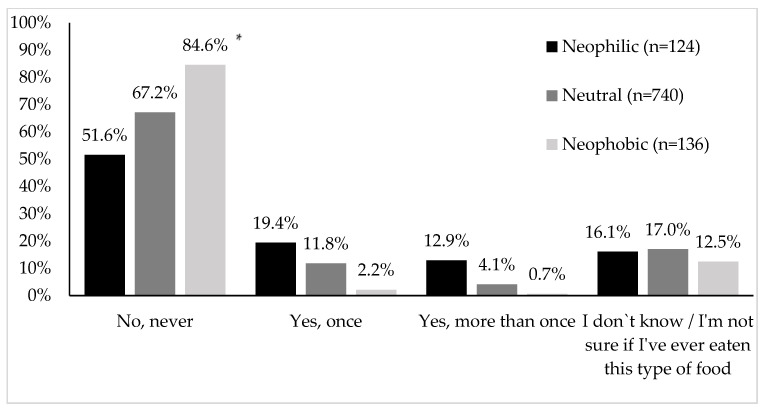
Willingness to try insects or insect-based food by food neophobia group. * Chi-square test; *p* < 0.0001.

Over 80% of study participants with high levels of food neophobia had never eaten insects or insect-based foods (Figure 1).

Less than half of respondents who had never eaten this type of food had a low level of food neophobia. The food neophobia score in the group of respondents who had never eaten insects or insect-based food was 41.30 ± 10.249. Among the respondents who had eaten insects or insect-based food at least once, 19.4% were respondents with a low level of food neophobia. Only 2.2% of respondents with high levels of food neophobia had eaten this type of food at least once in their lives. The level of food neophobia in this group was 35.44 ± 8.539. The lowest level of food neophobia was recorded in the respondents who declared that they had eaten insects or insect-based food more than once (32.60 ± 10.369). More than 12% of respondents in this group were people with a low level of food neophobia, and less than 1% had a high level. Among the study participants who were unsure whether they had ever eaten this type of food, 17% were people with an average food neophobia level. The level of food neophobia in this group was 39.44 ± 8.680.

### 3.3. Willingness to Try New Foods or Food Products

Most respondents said they buy new food products relatively quickly, although after some consideration (41.3%) (Table 3).

More than 20% of study participants indicated they were the first among their friends to buy new food products. A similar share of respondents indicated buying a new food product after some friends had already tried it. The fewest respondents said they were reluctant to buy new food products. Taking into account the level of food neophobia in the analysis, it was noted that respondents with the highest level of neophobia most often indicated the statements “I’m reluctant to buy new foods” (22.8%) and “I buy a new food product when most of my friends have already bought it and rated it positively” (18.4%). Respondents with the lowest level of food neophobia most often said that they were the first among their friends to enjoy buying new food products (25.0%) (Table 3). The differences were statistically significant at the level of *p* < 0.001.

### 3.4. Beliefs about Eating Insects

Most respondents agreed with the statement, “Some people would have allergic reactions to eating insects” (mean 5.08) (Table 3). Also, many respondents agreed that they were offended by the idea of eating insects and that eating insects is disgusting (both mean 4.87). Many respondents declared that eating insects makes them feel nauseous (mean 4.73) and ill (mean 4.72). Concerns were raised by some respondents about insects carrying harmful microbes (mean 4.69) or harmful toxins (mean 4.45) and the potential exposure to harmful chemicals and insecticides through eating insects (mean 4.42). On the other hand, study participants also concurred with the statement, “Insects contain high levels of high-quality animal protein” (mean 4.64). Fewer respondents agreed that insects are highly nutritious (mean 4.26). Respondents also agreed with statements indicating that farming insects for food require much less space than traditional animal farming (mean 4.84), is more efficient, and requires fewer resources than conventional animal farming (mean 4.57). Additionally, respondents concurred that insect farming generates less pollution and greenhouse gases than conventional animal farming (mean 4.53). The smallest number of respondents agreed with the statement that insects have a mild and rather pleasant taste (mean 3.86) and that they would rather not be friends with someone who eats insects regularly (mean 3.69) (Table 4).

The exploratory factor analyses were conducted using maximum likelihood extraction with varimax rotation (Table 4). The Kaiser–Meyer–Olkin measure verified the adequacy of the sample for analysis (KMO = 0.930), indicating that the choice of analysis and the number of factors were appropriate. Bartlett’s test of sphericity x^2^ = 9611.098, *p* < 0.001, indicated that correlations between items were high enough to perform the analysis. The results of the EFA of the 17 items made it possible to extract four factors. All factors were identified with an eigenvalue higher than the Kaiser criterion 1. The first factor’s eigenvalue is 7.079, which explains 41.64% of the variance. The second factor’s eigenvalue equals 2.705, which explains 15.913% of the variance. The third factor’s eigenvalue equals 1.056, which explains 6.21% of the variance. The fourth factor’s eigenvalue equals 1.002, which explains 5.90% of the variance. They explained 69.66% of the total variance. It has been arbitrarily assumed that the components of the factor are those variables that, after rounding, obtain absolute values equal to 0.5 or greater. The four factors were interpreted as follows: disgust (factor 1), risks (factor 2), general benefits (factor 3), and private benefits (factor 4) (Table 5).

The disgust factor encompasses items pertaining to the aversion and repulsion towards the idea of consuming insects, as evidenced by high loadings on statements reflecting feelings of nausea, illness, and disgust associated with eating insects. The risks factor comprises items concerning perceived risks associated with consuming insects, including concerns about infectious diseases, harmful toxins, and allergic reactions. The general benefits factor reflects beliefs concerning the broader societal and environmental benefits of insect consumption as a food source. Items loading on this factor underscore advantages such as the efficient utilization of resources, diminished pollution and greenhouse gas emissions, and the substantial value of insects as a source of proteins. The private benefits factor encompasses beliefs pertaining to personal advantages or benefits of consuming insects. Items with high loadings on this factor include statements regarding insects possessing a mild and rather pleasant taste and being highly nutritious. The reliability coefficient (α = 0.703) suggests a moderate level of internal consistency among these items, indicating that they measure the construct of private benefits with satisfactory reliability.

Including “food neophobia” in the analysis revealed a consistent pattern. Respondents with a high level of food neophobia scored significantly higher in the disgust and risks factors. Conversely, respondents with a low level of neophobia achieved the highest score in the general benefits factor (Table 6).

For the factor of disgust, a significant difference was observed between the three groups. “Neophobic” respondents had the highest mean score (5.95), indicating the strongest aversion to insects, followed by “neutral” (4.54) and “neophilic” (2.79). Similarly, for the factor of risks, there was a significant difference between the groups. “Neophobic” had the highest mean score (5.51), indicating the highest perceived risks associated with insect consumption. In terms of general benefits, a significant difference was also observed between the groups. “Neophilic” had the highest mean score (5.55), indicating the strongest belief in the general benefits of consuming insects, followed by “neutral” (4.56) and “neophobic” (4.23). Lastly, for private benefits, a significant difference was found between the groups. “Neophilic” had the highest mean score (4.71), indicating the strongest belief in the private benefits of consuming insects, followed by “neutral” (4.14) and “neophobic” (3.02).

### 3.5. Perception of Food Products Containing Insect Proteins

**Figure 2:**
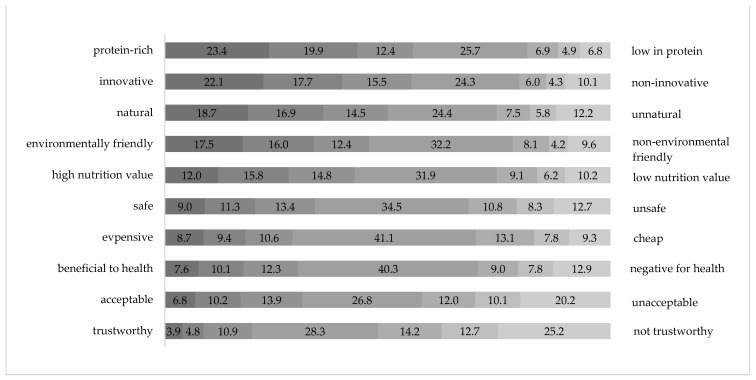
Perception of food products with added insect proteins on seven-point semantic differential scales, frequency distribution in percentages.

Respondents primarily perceived foods with added insect proteins as protein-rich, innovative, natural, and environmentally friendly (Figure 2). The respondents were least likely to perceive this type of food as trustworthy, acceptable, beneficial to health, and expensive.

Considering the respondents’ food neophobia level in the analysis, it was observed that individuals with a low level of food neophobia primarily perceived food containing insect proteins as rich in protein, innovative, environmentally friendly, and natural (see Table 7). Conversely, those with a high level of food neophobia primarily regarded this type of food as untrustworthy, unacceptable, unfavorable for health, and unsafe. Interestingly, there were no significant differences in the perception of expense across different levels of food neophobia.

### 3.6. Willingness to Eat Insects and Insect-Based Products

The respondents were asked to rate their willingness to eat food containing insects on a seven-point scale. It was observed that they were least inclined to eat food with visible whole insects, with a mean score of 2.45 (Table 8).

Respondents were slightly more inclined to consume food containing invisible insects, with a mean score of 2.70. The inclination to consume ground insect powder (e.g., insect powder in bread, pasta) and isolated insect protein (e.g., insect protein in a protein bar) was slightly higher, with means of 3.47 and 3.53, respectively. When analyzing respondents’ answers while considering food neophobia, it was discovered that among those categorized as “neophilic”, the inclination to consume isolated insect protein (e.g., insect protein in a protein bar) was significantly higher compared to other groups, with a mean score of 5.41. Conversely, in the “neophobic” group, this statement received an average score of 1.69. None of the statements among “neophobic” respondents achieved an average score exceeding 1.70. Respondents categorized as “neophilic” rated statements regarding willingness to eat whole insects, albeit invisible (e.g., chocolate-covered insects), and ground insect powder (e.g., insect powder in bread, pasta) above the midpoint of the scale, with scores of 4.28 and 5.27, respectively. Only the statement regarding eating visible whole insects (e.g., fried insects) was rated below this point, with an average score of 3.77 (Table 8).

## 4. Discussion

Our results confirmed patterns observed in the literature, including demonstrating that participants with food neophobia were older than neutral individuals and food neophiles [35]. Additionally, they had lower levels of education and income. In contrast, food neophiles were younger, better educated, had higher incomes, and had children in the household.

The highest-scored statements regarding beliefs about insects pertained to possible allergic reactions caused by consuming insects, disgust, and the belief that rearing insects for food requires much less space than rearing conventional livestock, reflecting one of the environmental aspects arising from the utilization of insects as a protein source. Despite the fact that one of the most prevalent beliefs is the feeling of disgust and aversion towards the idea of consuming insects [21,30,36], for Poles, the health risk associated with allergies was more prevalent. While the likelihood of allergic reactions to insects is generally low, perceived risk can significantly impact consumer behavior [37]. Individuals with a history of allergies or those with family members who have allergies may exhibit heightened caution when considering new foods, including insect-based products. The literature highlights potential risks linked to insect consumption, mainly arising from the possible presence of chemical contaminants like heavy metals and microbiological contaminants [38]. Certain insects have been documented to trigger allergic reactions through various routes, including inhalation, direct contact, stings/bites, and ingestion [14,39]. This link between allergic reactions to insect bites and the perception of insects as allergens in food can shape the overall perception of insects as potentially harmful or allergenic. Understanding and addressing these perceptions are crucial for promoting the acceptance of insect-based foods. The lowest-rated statements regarding beliefs about insects were related to taste, specifically “Insects have a mild and rather pleasant taste” and “I would rather not be friends with someone who eats insects regularly”. This suggests that respondents are skeptical about the taste of insects but do not hold a negative attitude toward individuals who consume them. There can be social stigma associated with eating insects, leading to concerns about being judged or ostracized by others. Beliefs about social acceptance and peer influence play a role in shaping attitudes toward insect consumption [22,40,41]. As revealed by factor analysis, the findings of our study on beliefs associated with eating insects align with previous research highlighting the influence of food neophobia on consumer perceptions of insect-based foods. Overall, our data suggest that individuals with higher levels of food neophobia tend to perceive greater disgust, risks, and lower benefits, both general and private, associated with insect consumption compared to individuals with lower levels of food neophobia. Our results are consistent with studies which also reported a link between food neophobia and attitudes towards insect consumption [15,21,27,42]. These studies emphasized the role of food neophobia in shaping individuals’ aversion to insects and their perceptions of associated risks and benefits. Moreover, our findings support the broader literature on food neophobia, which suggests that individuals with higher levels of neophobia tend to exhibit greater hesitancy towards novel or unfamiliar food items [43,44] Respondents categorized as neophobic were also less inclined to indicate that they were the first among their friends to try new foods and significantly more likely to admit that they had never consumed insect-based foods. The aversion to insects observed among neophobic individuals is consistent with their general reluctance to try new foods, as insects represent a non-traditional protein source in many Western cultures. The present findings are in line with the existing literature underscoring the significant impact of food neophobia on consumer perceptions and acceptance of novel food products [26,45]. Extensive research has consistently demonstrated that individuals with lower levels of food neophobia exhibit greater openness to experimenting with unconventional foods, including those incorporating alternative protein sources such as insects [26,28]. These individuals tend to view such foods favorably, emphasizing their nutritional richness, environmental sustainability, and alignment with natural dietary choices [21].

Our results show that neophobia affects the perception of food products with added insect proteins. Individuals’ varying levels of food neophobia shape their views on food containing insect proteins. Specifically, respondents with lower levels of food neophobia tended to perceive such food as rich in protein, innovative, environmentally friendly, and natural. Conversely, those with higher levels of food neophobia were more likely to perceive these products as untrustworthy, unacceptable, detrimental to health, and unsafe. Their perceptions are often influenced by concerns regarding safety, trustworthiness, and perceived risks associated with consuming unconventional food items [46]. Interestingly, our study revealed no significant differences in the perception of expense across varying levels of food neophobia.

The analysis of data regarding respondents’ readiness to accept insects suggests a mild preference among respondents for consuming insect-based products in forms where insects are less visibly identifiable. This is evidenced by the higher mean scores for food containing invisible insects, ground insect powder, and isolated insect protein compared to other forms. Such preferences may stem from a psychological barrier associated with the visual presence of insects in food, aligning with existing research on the role of disgust and aversion in consumer behavior towards insect consumption [3,11,13,26]. Greater food neophobia correlated with reduced willingness to consume insects, indicating a reluctance among individuals who are more neophobic to try insect-based foods. The analysis of respondents’ answers within the framework of food neophobia highlights significant differences in willingness to consume insects. “Neophilic” respondents, characterized by their openness to trying new foods, demonstrated a markedly higher inclination towards consuming isolated insect protein. Conversely, “neophobic” individuals exhibited a much lower willingness to consume insect-based products. This confirms the results that while consumers may be more receptive to insect-based products in processed forms, their acceptance of whole insects remains limited due to sensory aversions or cultural norms surrounding insect consumption. Sogari et al. [27] indicate that both food neophobia and disgust have a negative impact on the willingness to eat whole and processed insects and that food neophobia has a stronger association with the willingness to eat among individuals who consume insects compared to those who do not. Disgust was associated with lower self-reported willingness to eat insects, although this effect was not observed in actual behavior [47]. This suggests that while individuals may report feeling repulsed by the idea of eating insects, their actual behavior may not align with this self-reported aversion. As insects gain more recognition as a viable source of human food and an increasing number of insect-derived food items become available, it is commonly assumed that there will be a reduction in food neophobia [12]. Strategies aimed at mitigating neophobic responses and addressing negative perceptions through targeted marketing, educational initiatives, and sensory experiences may facilitate the broader adoption of insect-based foods in the future [12,48].

## 5. Conclusions

As insects continue to gain recognition as a viable food source, targeted marketing and educational initiatives aimed at mitigating food neophobia could play a crucial role in expanding the acceptance of insect-based foods. Strategies focusing on addressing negative perceptions and enhancing sensory experiences may be particularly effective for individuals open to insect consumption, potentially reducing reluctance and promoting the adoption of insect-based foods in the future.

The contrast between neophilic and neophobic attitudes underscores the need for targeted marketing strategies and product development approaches to meet the diverse consumer expectations within the rapidly expanding insect-based food market. These results underscore the importance of addressing food neophobia in promoting the acceptance of insect-based food products. Strategies aimed at providing education, exposure, and familiarization with insect proteins may be crucial in overcoming neophobic responses and fostering consumer acceptance of these sustainable and nutritious alternatives. Regarding beliefs about insects, our study found that while disgust and aversion are prevalent beliefs, Poles are more concerned about allergic reactions. Additionally, we identified a preference for consuming insect-based products in forms where insects are less visibly identifiable, suggesting a psychological barrier associated with the visual presence of insects in food. These findings highlight the importance of addressing food neophobia and tailoring strategies to address diverse consumer preferences in promoting the acceptance of insect-based foods.

## Figures and Tables

**Table 1:** Descriptive statistics of the sample.

Characteristics	Frequency	Sample (%)
Total	1000	100
Gender		
Male	470	47.0
Female	530	53.0
Age		
18–29	209	20.9
30–39	191	19.1
40–49	154	15.4
50–59	175	17.5
60 and over	271	27.1
Education		
Primary	21	2.1
Vocational	94	9.4
Secondary	481	48.1
Higher	404	40.4
Occupation		
Student	76	7.6
Full-time work	515	51.5
Part-time work/freelance work	96	9.6
Unemployed	79	7.9
Retired	217	21.7
Self-employed	51	5.1
Maternity/paternity leave	10	1.0
Parental leave	7	0.7
Household income		
Up to PLN 1000	43	4.3
PLN 1001–5000	299	29.9
PLN 5001–9000	364	36.4
Over PLN 9000	143	14.3
Refusal to answer	151	15.1
Assessment of the financial situation		
I do not have enough money for my basic needs	21	2.1
I live sparingly and have enough money for my basic needs	114	11.4
I live sparingly to put aside money for bigger expenses	613	61.3
I have enough money for everything without special savings	199	19.9
I am wealthy	20	2.0
Not specified	33	3.3
Number of people in household		
1	113	11.3
2	275	27.5
3	273	27.3
4	223	22.3
5 and over	116	11.6
Place of residence		
Rural	407	40.7
City up to 100,000	317	31.7
City 100,000–500,000	168	16.8
City over 500,000	108	10.8
Children under 18 years in household		
Yes	412	41.2
No	475	47.5
No answer	113	11.3

**Table 2:** Sample characteristics according to food neophobia group.

Variables	Food Neophobia				*p*-Value *
	Neophilic	Neutral	Neophobic	Mean ± SD	
Total	124 (12.4)	740 (74.0)	136 (13.6)	39.92 ± 10.126	
Gender					
Male	49 (39.5)	374 (50.5)	47 (34.6)	39.87 ± 9.165	<0.001
Female	75 (60.5)	366 (49.5)	89 (65.4)	39.96 ± 10.916	
Age					
18–29	35 (28.2)	161 (21.8)	13 (9.6)	37.28 ± 9.259	<0.001
30–39	18 (14.5)	159 (21.5)	14 (10.3)	38.94 ± 8.851	
40–49	24 (19.4)	116 (15.7)	14 (10.3)	38.57 ± 9.491	
50–59	22 (17.7)	136 (18.4)	17 (12.5)	39.25 ± 9.239	
60 and over	25 (20.2)	168 (22.7)	78 (57.4)	43.85 ± 11.369	
Education					
Primary	2 (1.6)	19 (2.6)	0	37.43 ± 6.577	
Vocational	6 (4.8)	72 (9.7)	16 (11.8)	41.63 ± 8.968	0.026
Secondary	52 (41.9)	356 (48.1)	73 (53.7)	40.49 ± 10.294	
Higher	64 (51.6)	293 (39.6)	47 (34.6)	38.97 ± 10.245	
Household income					
Up to PLN 1000	4 (3.2)	35 (4.7)	4 (2.9)	40.09 ± 7.476	0.030
PLN 1001–5000	26 (21.0)	222 (30.0)	51 (37.5)	41.48 ± 10.455	
PLN 5001–9000	53 (42.7)	269 (36.4)	42 (30.9)	38.75 ± 9.927	
Over PLN 9000	27 (21.8)	100 (13.5)	16 (11.8)	38.21± 10.969	
Refusal to answer	14 (11.3)	114 (15.4)	23 (16.9)	41.22 ± 9.225	
Assessment of the financial situation					0.004
I do not have enough money for my basic needs	1 (0.8)	17 (2.3)	3 (2.2)	40.52 ± 6.290	
I live sparingly and have enough money for my basic needs	6 (4.8)	91 (12.3)	17 (12.5)	42.18 ± 9.245	
I live sparingly to put aside money for bigger expenses	74 (59.7)	448 (60.5)	91 (66.9)	40.21 ± 10.474	
I have enough money for everything without special savings	40 (32.3)	140 (18.9)	19 (14.0)	37.22 ± 9.890	
I am wealthy	3 (2.4)	16 (2.2)	1 (0.7)	38.85 ± 7.652	
Not specified	0	28 (3.8)	5 (3.7)	43.33 ± 7.651	
Number of people in household					
1	10 (8.1)	77 (10.4)	26 (19.1)	43.18 ± 11.661	<0.001
2	33 (26.6)	185 (25.0)	57 (41.9)	41.17 ± 10.926	
3	32 (25.8)	216 (29.2)	25 (18.4)	39.00 ± 9.326	
4	35 (28.2)	171 (23.1)	17 (12.5)	38.33 ± 9.206	
5 and over	14 (11.3)	91 (12.3)	11 (8.1)	39.01 ± 9.089	
Place of residence					0.555
Rural	42 (33.9)	315 (42.6)	50 (36.8)	40.38 ± 9.364	
City up to 100,000	45 (36.9)	229 (30.9)	43 (31.6)	39.65 ± 10.524	
City 100,000–500,000	23 (18.5)	119 (16.1)	26 (19.1)	39.18 ± 10.805	
City over 500,000	14 (11.3)	77 (10.4)	17 (12.5)	40.11 ± 10.595	
Children under 18 years in household					<0.001
Yes	62 (50.0)	320 (43.2)	30 (22.1)	38.00 ± 9.351	
No	52 (41.9)	343 (46.4)	80 (58.8)	40.81 ± 10.082	
No answer	10 (8.1)	77 (10.4)	26 (19.1)	43.18 ± 11.661	

* *p*-values for chi-square analyses based on food neophobia group (*p* < 0.0001).

**Table 3:** Willingness to try new foods or food products including food neophobia.

Variables	Total	Food Neophobia (%) *		
	100%	Neophilic	Neutral	Neophobic
I buy a new food product relatively quickly, although after some thought	41.3	53.2	41.4	30.1
I buy a new food product when some friends have already tried it	21.5	18.5	22.6	18.4
I’m the first of my friends to like to buy new foods	20.3	25.0	21.4	10.3
I buy a new food product when most of my friends have already bought it and rated it positively	9.8	1.6	9.6	18.4
I’m reluctant to buy new foods	7.1	1.6	5.1	22.8

* Chi-square test; *p* < 0.001.

**Table 4:** Mean scores of insect eating variables.

Variables	Mean *	SD
Some people would have allergic reactions to eating insects	5.08	1.404
I am offended by the idea of eating insects	4.87	1.764
Eating insects is disgusting	4.87	1.811
Rearing insects for food requires much less space than rearing conventional livestock	4.84	1.514
The idea of eating insects makes me nauseous	4.73	1.879
The idea of eating insects makes me ill	4.72	1.850
Insects carry harmful microbes	4.69	1.490
Insects contain high levels of high-quality animal protein	4.64	1.495
Rearing insects as food is more efficient and requires fewer resources than rearing conventional livestock	4.57	1.518
Rearing insects for food generates less pollution and greenhouse gas than rearing conventional livestock	4.53	1.534
Insects contain harmful toxins	4.45	1.444
Eating insects would expose me to harmful chemicals and insecticides	4.42	1.520
Eating insects will increase risk of infectious disease	4.32	1.541
Insects are highly nutritious	4.26	1.553
It is unacceptable to eat insects in public	4.22	1.853
Insects have a mild and rather pleasant taste	3.86	1.402
I would rather not to be friends with someone who eats insects regularly	3.69	1.865

* A seven-point of scale—a rating of “1”—strongly disagree; a rating of “7”—strongly agree.

**Table 5:** Exploratory factor analysis of beliefs about eating insects.

	Factor and Loadings			
	Disgust α = 0.915	Risks α = 0.848	General Benefits α = 0.816	Private Benefits α = 0.703
The idea of eating insects makes me nauseous	0.850			
The idea of eating insects makes me ill	0.843			
Eating insects is disgusting	0.838			
I am offended by the idea of eating insects	0.746			
It is unacceptable to eat insects in public	0.698			
I would rather not to be friends with someone who eats insects regularly	0.572			
Eating insects will increase risk of infectious disease		0.745		
Insects contain harmful toxins		0.742		
Eating insects would expose me to harmful chemicals and insecticides		0.735		
Insects carry harmful microbes		0.730		
Some people would have allergic reactions to eating insects		0.573		
Rearing insects for food requires much less space than rearing conventional livestock			0.803	
Rearing insects as food is more efficient and requires fewer resources than rearing conventional livestock			0.792	
Rearing insects for food generates less pollution and greenhouse gas than rearing conventional livestock			0.774	
Insects contain high levels of high-quality animal protein			0.716	
Insects have a mild and rather pleasant taste				0.777
Insects are highly nutritious				0.595

**Table 6:** Mean scores for beliefs about eating insects *.

Factor			Food Neophobia						*p*-Value *
	Total		Neophilic		Neutral		Neophobic		
	Mean	SD	Mean	SD	Mean	SD	Mean	SD	
Disgust	4.51	1.53	2.79 ^a^	1.43	4.54 ^b^	1.32	5.95 ^c^	1.09	<0.001
Risks	4.59	1.16	3.76 ^a^	1.10	4.56 ^b^	1.06	5.51 ^c^	1.16	<0.001
General benefits	4.64	1.29	5.55 ^a^	1.10	4.56 ^b^	1.13	4.23 ^c^	1.36	<0.001
Private benefits	4.06	1.29	4.71 ^a^	1.09	4.14 ^b^	1.21	3.02 ^c^	1.31	<0.001

* ANOVA statistics; *p* < 0.001. ^a,b,c^ Means with the same letter are not significantly different from each other (Scheffé’s method; *p* < 0.001).

**Table 7:** Mean perception scores of food products with added/containing insect proteins on seven-point semantic differential scales.

Variables	Total		Food Neophobia						*p*-Value *
			Neophilic		Neutral		Neophobic		
	Mean	SD	Mean	SD	Mean	SD	Mean	SD	
Beneficial to health/unfavorable for health	4.08	1.664	2.98 ^a^	1.445	4.04 ^b^	1.550	5.33 ^c^	1.656	<0.001
Environmentally friendly/non-environmentally friendly	3.48	1.786	2.13 ^a^	1.391	3.52 ^b^	1.663	4.51 ^c^	1.985	<0.001
Natural/unnatural	3.51	1.917	2.15 ^a^	1.424	3.60 ^b^	1.804	4.31 ^c^	2.269	<0.001
Acceptable/unacceptable	4.38	1.833	2.96 ^a^	1.689	4.33 ^b^	1.684	5.97 ^c^	1.520	<0.001
Safe/unsafe	4.02	1.727	2.63 ^a^	1.490	4.04 ^b^	1.583	5.23 ^c^	1.755	<0.001
High nutrition value/low nutrition value	3.70	1.740	2.54 ^a^	1.478	3.74 ^b^	1.619	4.54 ^c^	2.025	<0.001
Expensive/cheap	4.01	1.596	4.14 ^a^	1.543	4.00 ^a^	1.514	3.96 ^a^	2.031	0.625
Protein-rich/low in protein	3.15	1.783	1.85 ^a^	1.338	3.30 ^b^	1.697	3.51 ^c^	2.076	<0.001
Trustworthy/not trustworthy	4.83	1.698	3.89 ^a^	1.749	4.73 ^b^	1.601	6.24 ^c^	1.279	<0.001
Innovative/non-innovative	3.28	1.864	2.04 ^a^	1.382	3.40 ^b^	1.744	3.75 ^c^	2.369	<0.001

* ANOVA statistics; *p* < 0.001. ^a,b,c^ Means with the same letter are not significantly different from each other (Scheffé’s method; *p* < 0.001).

**Table 8:** Willingness to consume insects and insect-based products including food neophobia *.

Variables	Total		Food Neophobia						*p*-Value **
			Neophilic		Neutral		Neophobic		
	Mean	SD	Mean	SD	Mean	SD	Mean	SD	
Visible whole insects (e.g., fried insects)	2.45	1.777	3.77 ^a^	2.114	2.46 ^b^	1.687	1.15 ^c^	0.631	<0.001
Whole insects, but invisible (e.g., chocolate-covered insects)	2.70	1.881	4.28 ^a^	2.097	2.71 ^b^	1.760	1.22 ^c^	0.841	<0.001
Ground insect powder (e.g., insect powder in bread, pasta)	3.47	2.080	5.27 ^a^	1.964	3.49 ^b^	1.927	1.68 ^c^	1.403	<0.001
Isolated insect protein (e.g., insect protein in a protein bar)	3.53	2.111	5.41 ^a^	1.808	3.55 ^b^	1.974	1.69 ^c^	1.412	<0.001

* A seven-point of scale—a rating of “1”—definitely unwilling; a rating of “7”—definitely willing. ** ANOVA statistics; *p* < 0.001. ^a,b,c^ Means with the same letter are not significantly different from each other (Scheffé’s method; *p* < 0.001).

## Data Availability

The original contributions presented in the study are included in the article, further inquiries can be directed to the corresponding author.
